# Supplementary material for: Predictable and stable epimutations induced during clonal plant propagation with embryonic transcription factor
Source: PLoS Genet. 2022 Nov 16;18(11):e1010479. doi: 10.1371/journal.pgen.1010479 (PMC9731469; doi:10.1371/journal.pgen.1010479)
Supplement: Attachment — Submitted filename: Reviewer-comments-PLOS_050922.docx [file pgen.1010479.s013.docx]

**Response to Reviewer’s comments**

**Manuscript PGENETICS-D-22-00513**

**We thank the three reviewers for their constructive comments. We have included all their suggestions and responded to their comments.**

Reviewer #1: 

**Major:**
One of the themes of this paper is that the phenotypic and molecular imprints in regenerated plants were independent of the TF used for somatic embryogenesis. The paper was mostly focused on the LEC2 line, which I think is not a problem; but, in particular for the early senescence and reduced biomass of RO plants; it should be clarified if this was also observed in RKD4 plants? The RO-RKD4 plant in Fig 1B looks like the control (?) and the data in Fig1C-D is only for LEC2? Can the data for RKD4 be presented too, or is this phenotype specific for LEC2?

**The early senescence and reduced biomass phenotypes were only observed in Ws-2 plants regenerated from roots using LEC2 (RO-LEC2). We did not find evidence for early senescence in any of the Ws-2 plants regenerated with RKD4. We have revised the results section to clarify this point.**

**Minor:**
- it would be good to title the Results section “Results”!
**We have corrected the text in the revised manuscript.**

- please clarify if G0 is the regenerated generation (page 4) (I assume so but it is not stated)

**The G0 plants are the regenerated generation. These plants were propagated by selfing for three constitutive generations, combining seeds from ten randomly selected plants per line, to produce G1, G2 and G3 progenies. We have now revised the corresponding results section to make this clearer.**

- page 4 it says 4/5 RO plants Fig1C-D - but there are 10 RO plants in Fig1C???

**We apologise for the mistake, the correct sentences should be: “Most RO-LEC2 lines (6/10) had reduced biomass, smaller leaf area and senesced early (Fig. 1C-D)”. We have revised the results section accordingly.**

- The conclusion on Page 4 is that tissue of origin is the major determinant of phenotypic variation. I agree the phenotypes are impressive. But this conclusion seems too broad as the data are only for select phenotypes, I’d suggest revising to something more modest. (“a major contributor… to the phenotypes we measured”). Also, as above in the Major comment, Fig1C-D only includes data for LEC2, please include the data for RKD4 to support this statement or how do we know there isn’t a strong tissue x TF effect?

**Thank you for the suggestion. According to the reviewer comments we have revised the result section to: “Collectively, our data suggest that the tissue of origin employed for regeneration and the regeneration protocol could contribute to phenotypic diversity in the clonal plants and their progenies, thus pointing to the long-term inheritance of somatic epigenetic states in the sexual progeny of clonal plants”**

- page 5 (or in methods) please state how many biological replicates were used for RNA-seq. Looking at the supp tables, does one group (WT-root) have only 2 replicates? (on a side note, why still using Tophat!... 14 years old now)
**For RNA-seq we used 3 replicas in all samples, however we had to discard two libraries due to low quality. We have revised the Methods section to clarify this point:
“RNA-seq libraries from samples; Control-LEC2 root 2 and Control-RKD4 root 3, were discarded for further analysis due to bad quality”.**

- In the discussion about DEGs on page 5 it would be helpful to include the number(s) of DEGs in text when highlighting that some contrasts have more than others.
**We have included the number of DEGs in the revised text.**

- Fig 2C, D and E fonts are too small to read.
**In the revised manuscript we have rearranged Figure 2 so panel C is bigger and we have increased the font size in the nodes of the networks of panels D and E.**

- Is Fig2F RNA-seq (or eg qPCR)? Are the differences statistically significant, please add stats to this figure?
**Figure 2F values represent log2(Fold Change) of RNA-seq normalised counts, we have included that in the figure legend. All four genes are upregulated in a statistically significant way in leaves from RO plants but none of them is in leaves from LO plants. We have included the adjusted p-values for each of the gene:**

- **EDS5 (WS2_G0024164, AT4G39030): p = 0.00015 (***)**
- **ICS1/EDS16 (WS2_G0007317, AT1G74710): p = 0.00706 (**)**
- **PBS3 (WS2_G0025579, AT5G13320): p = 0.00036 (***)**
- **SARD1 (WS2_G0007214, AT1G73805): p = 0.00240 (**)**

- Please explain the axis categories in Fig3-S2 more clearly. “DMRs” = all DMR loci? But what does control, LO-G2 etc refer to; are these the DMRs in each genotype or is this all regions with methylation data coverage?
**DMRs in Figure 3 supplement 2B represent DMRs identified in an all-against-all analysis, as in figure 3A. Control, LO_G2 and the other samples represent the regions identified as methylated (by Methylscore) in each of the samples that are then used to compute DMRs. We have clarified this in the figure and in the legend.**

- Page 13 “...although they were faithfully inherited maternally (Fig4A)” should be Fig4B
**Thank you, we have corrected the text in the revised manuscript.**

- Is the Ws assembly available somewhere?
 **The *de novo* genome assembly for Ws-2 has been submitted at the European Nucleotide Archive (ENA) under BioProject PRJNA819891**

Reviewer #2: 

1. The study stops short of demonstrating a causal relationship between the two DMRs and the senescence phenotype. Is there are evidence in the Col-0 that genetic mutations in the genes flanking the DMRs cause senescence / disease resistance phenotypes?
 **We agree with this point- our study does not demonstrate the direct cause of the early senescence phenotype commonly observed in RO-LEC2 plants. We are not aware of a report linking PRE1/BNQ1 with senescence / disease resistance phenotypes in Arabidopsis. However, a *dgr2* T-DNA insertion mutant (Salk_125079) displays defects in root growth and has an early senescence phenotype (Gao et al., 2012).**

2. The authors state the leaf phenotypes of LEC2-RO plants are associated with genome-wide DNA hypomethylation, which resembles the methylation profile of these regions in roots from control plants. However, roots do not necessarily accumulate increased levels of SA – if anything they accumulate much lower levels of SA than leaves. (see e.g. Bagautdinova et al. Int J Mol Sci. 2022 Feb; 23(4): 2228). Can the authors provide an explanation why the root-derived epigenetic state in leaves results in SA hyperaccumulation, while the same epigenetic state in roots doesn’t?
 **The increased levels of SA in leaves of regenerated LEC2-RO plants may be a consequence of the transcriptional mis-regulation of defence and senescence pathways. In wild type roots, these pathways may have extra layers of regulation in addition to DNA methylation.**

3. Based on the transcriptome analysis of LEC2-RO plants, the authors assume that the sae epialleles control both senescence and SA hyperaccumulation. Can this be confirmed by the phenothpes of the F2 segregants? In other words, does the senescence phenotype co-segregate with increased SA levels and/or defence-related gene expression?

**We did not perform SA nor transcriptome measurement on F2 segregants because the early senescence phenotype found in Ws-2/LEC2-RO regenerants correlated with an increase in SA levels in leaves (Fig 2G).**

4. Related to the above points, total SA levels (free SA and SA glucoside) were quantified by the ADPWH-lux biosensor, which doesn’t differentiate between free SA and glycosylates forms of the SA. This is somewhat unfortunate as a disturbed balance between free and conjugated SA might explain the senescence phenotypes. It would be great if the authors could address this point in the Discussion and/or provide extra data where possible.

**Unfortunately, we measured SA levels with an ADPWH-lux biosensor, which represents total SA levels in leaf (free and conjugated). We have acknowledged in the manuscript that free SA and conjugated SA could have helped to explain the senescence phenotype.**

5. Top of 14: “4F [will change to G])”. Is this a left-over comments from the authors’s own revisions?
**Thank you, we have corrected the text in the revised manuscript.**

6. Figure 4E the different DNA methylation categories would benefit from a bit more explanation than the somewhat cryptic information provided in the legend.
**We have expanded the figure legend to provide a better explanation of Figure 4E**

7. Some of the sequencing tracks of the supplemental figures painfully reminded me to upgrade my reading glasses as they are labelled by very small letter fonts.
**In the revised manuscript we have increased the font size in figure 4 supplements 3 & 4.**

Reviewer #3: 
This is a well-written and comprehensive manuscript that describes heritable variation associated with the tissue from which clones are produced. The thorough analysis of sequence and epigenome data supports the conclusions that heritable variation is based - at least in significant part - on pre-existing patterns in the originating tissues. A reasonable hypothesis is advanced on the basis of the phenotype(s) observed over generations. The manuscript is very well done.

**Thank you for the positive comments.**
